# Supplementary material for: Are carbon emissions trading and green financial instruments synergistic? -Comprehensive quantitative research based on content analysis
Source: PLoS One. 2024 Mar 7;19(3):e0298601. doi: 10.1371/journal.pone.0298601 (PMC10919601; doi:10.1371/journal.pone.0298601)
Supplement: S1 Data — (ZIP) [file pone.0298601.s001.zip › Data/地级市Z_F工作报告.zip!/╣π╬≈/└┤▒÷╩╨/└┤╫╘╣1⁄2╓┌║┼í╛┬φ┐╦╩2╛▌═°í┐_2017.docx]

各位代表：

现在，我代表市人民政府，向大会报告工作，请予审议，并请各位政协委员和其他同志提出意见。

2016年工作回顾

过去的一年，面对经济下行、增长趋缓的严峻形势，在自治区党委、政府和市委的正确领导下，市人民政府团结全市各族人民，深入学习贯彻习近平总书记系列重要讲话精神，着力抓好稳增长、促改革、调结构、惠民生、防风险各项工作，全市经济社会平稳发展。实现地区生产总值589亿元，增长3.9%；规模以上工业增加值增长1.8%；固定资产投资370.9亿元，下降17.4%；财政收入49.6亿元，下降0.9%，非税收入占比下降3.9个百分点；社会消费品零售总额增长9.7%；服务业在生产总值中的比重上升到37.6%，比上年提高1.2个百分点；城镇新增就业1.07万人；居民消费价格总水平上涨2%，低于控制目标1个百分点；城镇居民人均可支配收入增长7.0%，农村居民人均可支配收入增长9.2%；金融机构存款余额增长14.9%，贷款余额增长13.1%。

尽管去年全市经济低位运行，但是市人民政府毫不松劲，不等不靠，担当作为，以“6个年”活动和招商引资大行动为抓手，立足于稳，着力于进，打基础、兴产业、利长远，全力推动经济“止跌回稳”，朝着走出困境的方向稳步发展，展现出蓄势待发、拨云见日的良好势头。

一、改革创新，实现突破瓶颈、扬帆起航

以改革创新重拳，突破制约瓶颈。一是推进“一网两管”建设，突破降成本体制制约。以实施国家售电侧市场改革试点为契机，大力推进大工业区域电网建设，预计上半年可通电调试运行，供电价格将明显下降。加快国家环境服务业试点建设，河南工业园东区集中供热项目基本建成，下游用热企业超过30家，来宾高新区集中供热项目稳步推进，集中供热可大幅节省用热成本。启动实施工业大用户天然气直供管道项目，用气成本可节省25%左右。落实降成本38条措施，为企业减负4.3亿元。二是推进“三区”改革，突破管理体制障碍。实施以简政放权为核心的市与城区、来华管理区（来宾高新区）、河南工业园区管理体制改革，下放事权70项，调整下划编制和人员485个，进一步明晰市与城区、管理区、园区的权责关系，提高管理运行效率。同时，将兴宾区凤凰镇、桥巩镇、八一街道、来华街道和凤凰工业园成建制委托给来华管理区（来宾高新区）管理，将兴宾区迁江镇成建制委托给市河南工业园区管理，利用托管乡镇的新优势，进一步做大做强园区经济。三是推进项目工作机制改革，突破项目建设瓶颈。针对重大项目落地难、推进慢、储备少等问题，成立了市重大项目推进办公室，建立协调机制，集中优势资源，打好项目建设大会战。四是推进招商理念转变，突破招商引资困境。建立招商项目快速落地和跟踪服务机制，实施招商激励政策，开展大招商、招大商、招强商，新签约正式协议项目94个，其中工业项目59个，签约规模395亿元，增长12.6%，其中全口径实际利用外资1.42亿美元，增长18.3%，一批重大项目实现落地开工。五是推进产业优化布局，突破区域协同发展壁垒。启动建设跨县域的来宾市三江口港产城新区，按照港产城一体化融合发展模式，建设集产业融合发展、科工贸为一体的大型港产城园区，带动和辐射县域产业发展，打造经济增长新一极。六是推动企业改制，突破企业发展困局。推动广铁公司加快改革改制步伐，永鑫、博宣、博华等糖业公司重组改制稳步推进，促进企业恢复生产、焕发生机。开展服务企业活动，累计帮扶重点企业59家，协调解决困难和问题152个。全市新增高新技术企业7家，总数达到14家。加大筹融资工作力度，化解了债务风险。

二、产业发展，奋力爬坡过坎、推动转型

以振兴产业为重点，着力开展“工业提质年”“农业提效年” “服务业提升年”活动，实体经济进一步发展。在工业方面，全市规模以上工业总产值526.8亿元，增长3.6%。传统产业二次创业初显成效，来宾银海铝二期等13个传统产业项目实现开竣工；新兴产业加快发展，福美新材料等21个项目开竣工，广西（来宾）现代农机装备产业园筹建工作进一步加快，新兴产业完成产值72亿元，增长12%。园区建设稳步推进，完成园区基础设施投资4.2亿元，园区工业总产值436.9亿元，武宣县工业园区成为我市县域首个突破百亿元产值的工业园区。在农业方面，农林牧渔业总产值增长3%。实施“粮安工程”，粮食产量81.6万吨；落实“双高”糖料蔗基地41.2万亩，占任务的103%，糖料蔗单产提高2%；桑园面积达59万亩，增长1.1%；水果面积72万亩，增长14.5%；木材产量231.6万立方米，增长26.2%；肉类总产量15.29万吨；水产品产量7万吨，增长5.1%。桂中治旱一期工程累计完成投资22.1亿元，占概算总投资的83.5%，二期工程累计完成投资5.95亿元；完成水利投资17.38亿元，除险加固病险水库92座，新增恢复改善灌溉面积10万亩，推进节水灌溉31万亩。年内建成自治区级现代特色农业（核心）示范区4个、市级11个、县级10个、乡镇级9个，目前全市有自治区级示范区5个、市级11个。全市土地流转53.3万亩。新增农民合作社366家、家庭农场103家。农村土地确权进度排全区前列。农业耕种收综合机械化水平达55.3%。新增“三品一标”认证10个，武宣县成功创建广西农产品质量安全县。在服务业方面，服务业增加值增长7.1%。交通固定资产投资完成48.3亿元。公路总里程7011公里，新增451公里；公路、水路货运周转量分别增长5.9%、12.4%；港口吞吐量连续5年超1000万吨。商贸物流服务功能不断提升，消费环境逐步优化，社会消费品零售总额159.1亿元。实施“电商来宾”工程取得初步成效，电商企业（网点）发展到352个，预计全年交易额35亿元，忻城县获商务部2016年电子商务进农村综合示范县，象州县电子商务实现县、乡、村三级服务网络全覆盖。全域旅游稳步发展，金秀县获列为第二批国家全域旅游示范区创建单位，全市接待游客1809万人次，增长17.3%，旅游总消费133.8亿元，增长31.5%。文化、金融、保险、养老等服务业加快发展。在县域产业发展方面，通过以城带乡，城乡互动，县域经济稳步发展。兴宾区着力打造百里现代农业旅游观光示范带，生产总值增长4.5%；象州县积极融入西江经济带建设，生产总值增长1.8%；武宣县突出抓好园区经济发展，生产总值增长5.1%；忻城县一手抓扶贫，一手抓旅游，生产总值增长3.5%；金秀县成功创建并利用“广西特色旅游名县”品牌发展生态旅游，生产总值增长3.2%；合山市全力脱贫攻坚，加快产业转型，生产总值增长2.0%；来华管理区（来宾高新区）重点发展高新技术产业和先进制造业，规模以上工业总产值增长11.1%；河南工业园区重点推进铝电一体化发展，规模以上工业总产值增长4.9%。

三、精准脱贫，全力集中攻坚、首战告捷

落实“一帮一联”，补好脱贫短板。实施“脱贫攻坚年”活动，全面完成贫困户入户登记，全市共安排帮扶干部2.2万人，结对帮扶贫困户7.31万户，实现帮扶全覆盖。加大产业扶贫力度，实现所有贫困村扶贫产业全覆盖，共有3.9万贫困户15.1万人受益。加大扶贫资金投入，完成易地扶贫搬迁投入5亿元，发放扶贫小额信贷8.44亿元，财政扶贫资金投入8.59亿元，比上年投入翻两番以上。完成脱贫“双认定”8.4万人，超额完成自治区下达的年度任务，预摘帽的合山市和全市42个贫困村通过自治区的核验。

四、基础设施，做到城乡统筹、提升完善

实施“项目建设落实年”和“西江经济带建设推进年”活动，项目拉动能力稳步增强。实施重大项目240个，其中新开工42个、续建95个、竣工投产36个。149个西江经济带项目完成投资66.91亿元。天丞铝轮毂等30个产业项目实现开竣工。梧柳高速来宾段、柳南高速来宾段改扩建工程加快建设，象州港区猛山作业区一期工程、合山港建成运营。创新投融资体制，新增融资99亿元。推进新型城镇化建设，城乡面貌展现新形象。国家新型城镇化综合试点落实33项具体任务，安排农民工等新市民首次购房补助2299万元，新增城镇人口4万人，城镇化率达42.2%，提高1.5个百分点，全区新型城镇化综合试点现场会在我市召开。开展了海绵城市、地下管廊等10项专项规划编制。启动城北新区完善提升配套工程，实施项目20个，完成投资4.9亿元。推进旧城区23条道路改造，加快全国无障碍环境示范市建设。特色村镇建设取得实效。启动实施城市社区建设大会战。城市管理水平不断提升，智慧城市建设加快推进。实施城乡绿化工程，市城区绿化覆盖率40%；完成植树造林面积26万亩，森林覆盖率51.8%，其中金秀县森林覆盖率居全区第1位，我市荣获“国家森林城市”称号；“美丽来宾·生态乡村”建设取得阶段性成效，完成471个自治区级示范村屯和4525个一般村屯绿化建设，完成346处饮水安全工程和14处饮水净化示范项目建设，完成71个行政村、513个自然屯道路建设和1126公里屯内道路硬化，生态乡村建设满意度排全区第3位。加强环境保护，空气质量优良天数达324天，饮用水源地水质达标率100%，万元生产总值能耗预计下降5%，二氧化硫、氮氧化物、化学需氧量和氨氮排放量均控制在自治区下达指标任务以内；推进12个城镇污水生活垃圾处理设施建设，市城区生活垃圾无害化处理率达100%，污水处理率在87%以上，城乡环境进一步改善。

五、社会事业，突显亮点纷呈、和谐有序

强化底线思维，民生福祉和社会事业更富成效。荣获“全国双拥模范城”“全国无偿献血先进市”等称号。完成53个村级公共服务中心建设，顺利通过国家基层文化服务中心建设试点验收。完成义务教育均衡发展“国检”“区检”目标任务，成为广西第一个整市通过义务教育均衡发展省级评估的设区市；开展消除市城区“大班额”攻坚，筹措10亿元新建、改扩建城区义务教育学校20所，已建成5所；学前教育扩容增量，新增普惠幼儿园130所；加快普及高中阶段教育，筹措15亿元启动城区普通高中规划建设；组建来宾职业技术学院；市城区在校大学生和中职生双双突破万人。举办各类文化体育活动2万多场次，实施83个行政村通有线广播电视工程，群众精神文化生活丰富多彩。全面两孩政策稳步实施，全市生育水平保持平稳；医疗卫生体制改革稳步推进，基本公共卫生服务体系建设居全区中上水平，新农合参合率99.3%。建成保障性住房4397套，分配入住4000套，完成农村危房改造1万多户。城镇登记失业率3.4%，低于控制目标0.7个百分点；城乡居民基本养老保险参保率95.3%；城乡居民享受最低生活保障154.5万人次，实现应保尽保。社会治理创新扎实推进，进一步完善“天网”工程建设，深化网格化管理，社会治安综合治理成效显著，刑事案件下降15.4%，治安案件下降7%，城区“两抢”案件下降17.7%，群众安全感位居全区第3位。调结“三大纠纷”案件407起，调结率90%；人民调解案件调处成功率达96.2%。信访总件数及人次分别下降7.7%和18.4%。应急管理体系进一步完善，应急保障能力进一步提升。安全生产形势持续稳定，各类安全事故数与去年持平，死亡人数下降2%。加强食品药品监管，食品农产品抽检合格率达98%以上，药品抽检合格率达98.8%以上。

六、政务服务，力求简政放权、提高效能

千方百计营造务实高效、风清气正的政务环境。扎实做好简政放权工作，市本级行政审批事项减少到228项，再精简6.9%。办结行政审批服务事项52万件，按时办结率100%。坚持依法行政和民主决策，推行政务公开。全面落实市委的决策部署，认真执行市人大及其常委会决议、决定，主动接受市人大及其常委会的法律监督和市政协的民主监督，人大代表议案和政协委员提案办结率均达100%。扎实开展“两学一做”学习教育，政府履职能力进一步提升。认真落实党风廉政建设主体责任，严格遵守中央八项规定精神，不断加强作风建设，坚决查处发生在群众身边“四风”和腐败问题，党风廉政建设和反腐败工作取得新成效。

一年来，军民融合、机关事务管理、广播电视、新闻出版、机构编制、法律援助、民族宗教、外事侨务、社会科学、政策咨询、地方志、残疾人、科协、工商联、工会、共青团、妇联等工作各显特色，统计、审计、供销、气象、地震、人防、工商、质监、消防、档案、保密、老龄、金融、税务、石油、电力、烟草、通信、邮政等工作各具亮点，取得了新的成绩，为经济社会发展作出了积极贡献。

各位代表，过去一年，两届政府顺利完成接力棒交接，积极化解经济下行压力，夯实了经济基础，激发了社会活力，为“十三五”发展开好头、起好步，取得的成绩来之不易，经验弥足珍贵。这主要得益于自治区党委、政府和市委的正确领导，得益于市人大和市政协的监督指导，得益于社会各界的鼎力支持，是全市各族人民同心同德、奋力拼搏的结果！在此，我代表市人民政府，向全市各族人民，向人大代表、政协委员，向驻来宾的中直、区直部门和人民解放军、武警官兵，向各民主党派、无党派人士、工商联、人民团体，向所有关心和支持来宾发展的社会各界人士，表示衷心的感谢和崇高的敬意！

过去一年，尽管我们集中力量突破瓶颈、打牢基础，但目前来宾仍是广西14个设区市中经济下行压力最大的，我市经济还没有真正走出困境，还需下一番苦功夫，过一段苦日子。我市经济发展中存在的最大问题就是“工业不强，农业不优，商旅不旺”，遇到的最大困难就是“项目不多，财力不足，债务不轻”。我们要擦亮眼睛看清当前面临的最大问题和困难。习近平总书记指出：“有问题不可怕，可怕的是不敢直面问题，找不到解决问题的思路。我们既要有分析问题的智慧，更要有采取行动的勇气；遇到了困难，不要埋怨自己，不要指责他人，不要放弃信心，不要逃避责任，而是要一起来战胜困难。历史是勇敢者创造的，让我们拿出信心，采取行动，携手向着未来前进。”（见注释1）我们一定要勇于担当，壮士断腕，保持定力，精准施策，不忘初心大兴产业，继续前进走出困境。

2017年目标任务

2017年是党的十九大召开之年，也是我市建市15周年，做好今年的政府工作，影响深远，意义重大。要坚持“明大势”，从国际国内形势分析，世界经济逐渐复苏，国内深入推进供给侧结构性改革，着力振兴实体经济，努力扩大消费，促进房地产市场平稳健康发展，经济增长仍保持较强动力。自治区第十一次党代会提出着力营造“三大生态”、奋力实现“两个建成”的宏伟目标，将出台一系列的稳增长、促发展、补短板等政策措施，给予来宾更有利的支持和释放更多的红利，我们要抓住新机遇、用好新政策加快来宾发展。

从我市发展的形势分析，去年，我市着力理清思路、推进改革、突破瓶颈，制定实施的改革措施，今年将逐步释放红利，为加快走出困境聚集积极能量。建设“一网两管”逐步发挥效益，将带动传统产业重焕生机，将成为我市招商引资的金字招牌和撬动产业发展的新杠杆；启动三江口港产城新区建设，将助推我市打造沿江经济发展新优势；获批国家环境服务业改革试点城市，将加快循环经济发展；全面加快新型城镇化建设步伐，城市功能不断完善，人气不断聚集；桂中治旱、“双高”糖料蔗基地、现代特色农业（核心）示范区等重点农业工程不断推进，为我市农业发展注入更大活力；服务业“四大集聚区”加快形成，“桂中商埠”集聚效应进一步显现；大力实施创新驱动战略，大众创业、万众创新成为潮流，新经济、新业态、新模式不断涌现；一批招商引资项目相继落地投产，将为经济发展提供更多支撑，聚焦产业、大兴产业已经成为来宾走出困境的重要共识，等等。这些有利条件表明，我市仍然具备保持经济较快增长的基础和条件，经济企稳向好的势头正在显现，我们走出困境将曙光在前、胜利在望。

今年政府工作的总体要求是：全面贯彻落实党的十八大和十八届三中、四中、五中、六中全会及中央、自治区经济工作会议精神，深入学习贯彻习近平总书记系列重要讲话精神和治国理政新理念新思想新战略，围绕自治区第十一次党代会、市四次党代会重大部署，按照市委工作会议提出的“明大势，抓重点，守底线，用干部，善作为”总要求，坚持稳中求进工作总基调，坚持以提高发展质量和效益为中心，以推进供给侧结构性改革为主线，以“做大产业、做强工业、做精招商、做实项目”为重点，狠抓脱贫攻坚，着力稳增长、促改革、调结构、惠民生、防风险，以建市15周年加快发展为契机，为推动来宾经济尽快走出困境、社会更加和谐发展、人民群众幸福安康打下更坚实的基础，以优异成绩迎接党的十九大胜利召开。

今年经济社会发展主要预期目标是：地区生产总值增长7.5%左右；规模以上工业增加值增长7.5%，固定资产投资增长15%，财政收入增长5%，社会消费品零售总额增长10%，居民人均可支配收入实际增长7.5%，居民消费价格总水平涨幅控制在3%左右。

我们要积极适应经济发展新常态，把握发展的新趋势、新要求，以更大的胆识推进改革，以更高的智慧加快创新，以更强的措施促进发展，在确保“稳”的前提下，努力在“进”上取得重大突破，全面完成上述发展目标任务。

围绕我市“十三五”规划目标和实现全面小康监测指标，我们一定要理清思路，突出重点，明确任务，做到攻坚克难谋发展，稳中求进谱新篇。要坚持“抓重点”，突出“做大产业、做强工业、做精招商、做实项目”重点。做大产业就是要利用资源优势，按照产业脱贫的要求，加快一二三产业融合发展，调整优化农业产业结构，提高农产品附加值，加快工业转型升级，促进服务业做大做旺，扩大消费规模，提升产业整体竞争力。做强工业就是要重点推动传统产业转型升级，加快发展壮大新兴产业，加大科技创新力度，促进企业做强做优，提高工业发展质量和效益。做精招商就是要以产业基础和资源优势为重点，紧盯大商、强商、实商，有目标、有重点、有计划实施精准招商，提高招商引资实效。做实项目就是要超前谋划，完善项目前期工作；要优化程序，促进项目落地开工；要跟踪服务，推动项目竣工达产；要完善机制，形成项目建设合力。具体要着力抓好以下九个方面的重点工作和主要任务。

一、着力实施供给侧结构性改革，提高经济发展质量

围绕“五大任务”和“两个重点”，全力推进供给侧结构性改革。去产能方面，引导小煤矿减压产能20万吨以上；实行资源共享、产业重组，帮助企业恢复生产、走出困境，加快清理“僵尸企业”。去库存方面，落实好购房补助政策，继续推行以购以租代建等模式，扩大商品房销售，争取商品住宅库存去化周期保持在合理区间。去杠杆方面，鼓励企业直接融资，争取直接融资占融资总额的比重超过20%。加强财税工作，强化政府性债务管理，防范债务风险。降成本方面，加快“一网两管”建设，破解企业生产成本高的难题；落实好结构性减税措施，实现企业制度性交易成本和生产经营性成本“双下降”。补短板方面，加大对脱贫攻坚、基础设施、科技创新、公共服务、民生事业等发展短板的扶持力度。农业供给侧结构性改革方面，进一步优化农业产业、产品、经营、动能结构，提高农业综合效益和竞争力。服务业供给侧结构性改革方面，按照个性化、定制式理念，加快发展旅游、文化、体育、健康、养老等幸福产业，打造一批现代服务业集聚区，全面提升供给服务水平。

二、着力推动转型升级，促进工业率先走出困境

突出工业龙头，促进转型升级，加快建设新兴现代化工业城市，全市规模以上工业总产值增长11%。

攻坚克难做优传统产业。优化服务激活存量，创新服务机制，抓好服务企业工作，落实稳增长、降成本措施，推动存量企业复产达产、做强做优。抓好项目扩大增量，以售电侧市场改革试点为突破口，加快铝电一体化进程，确保大工业区域电网年内通电运行，加快银海铝业二期、工业大用户天然气直供、河南工业园东区和来宾高新区集中供热等重点项目建设，重点发展黑色金属冶炼产业、新材料深加工和铝精深加工产业。抓好改革增添活力，推动企业兼并重组，加快广铁公司改革改制步伐。传统产业产值增长10%。

千方百计做大新兴产业。重点培育发展碳酸钙产业，推动年产50万吨碳酸钙粉料加工、年产20万吨钙化物等10个碳酸钙项目尽快实现开竣工，努力打造来宾百亿元碳酸钙产业。支持福美公司MCM生态材料循环经济产业园建设和装配式建筑产业发展。加快培育发展汽车（机械）配件产业，承接柳州汽车产业，重点推动年产500万件汽车橡胶件和中首信、国拓公司等现代农机装备制造、纳米材料等一批先进制造业项目建设。充分发挥美国斯坦福大学终身教授、世界纳米技术研究顶级专家、广西科技师范学院客聘教授崔屹教授作为来宾家乡人的优势，争取打造广西（来宾）纳米技术产业基地。与自治区农机局等厅局合作，加快广西（来宾）现代农机装备产业园建设。通过自治区农机局申请，争取“2017年中国甘蔗机械化博览会”在我市举办。加快延伸发展茧丝绸产业，推动与广东、浙江等地丝绸纺织产业集团、协会的战略合作，延伸茧丝绸产业链。新兴产业产值增长12%以上。

集中力量做强工业园区。积极创建国家级的热电联产循环经济生态工业园，工业大用户天然气直供项目年内开工建设，河南工业园区和来宾高新区的供热管道等项目年内竣工运行。积极创建百亿元工业园区，着力推进广西（来宾）现代农机装备产业园，兴宾区红河集中区和小平阳碳酸钙产业园区，象州汽配、新能源和染整业产业园，武宣碳酸钙产业园，忻城思练石材产业园，合山循环经济综合利用基地，合山忻城碳酸钙产业合作园区，金秀桐木、头排扶贫产业园等专业园区建设。象州县工业园区总产值要超88亿元，明年力争突破100亿元；河南工业园区工业总产值超180亿元，明年力争突破200亿元；来华管理区（来宾高新区）工业总产值超45亿元，明年突破50亿元。积极推进和完善园区管理体制改革，充分释放来华管理区（来宾高新区）和河南工业园区发展潜能，聚焦主业，做强工业，做到产城融合、园镇同建、城乡一体。2017年园区基础设施投资完成4.5亿元以上，园区工业总产值完成480亿元以上，增长10%。

加快步伐做实港产城融合。港产城一体化发展是实现工业现代化的重要途径。加快三江口港产城新区建设，完成新区总体规划编制，创新园区开发建设模式。制定完善新区配套政策措施，推进新区市场化、公司化体制运作模式建设，重点加快石龙片区建设，做好高安、金鸡、黄茆、马坪片区启动建设的前期工作，加快推进桐木、头排作为拓展园区的建设，配合自治区人民政府和自治区水产畜牧局，积极做好广西黔江示范牧场和广西武宣种畜场下放管理职能的各项工作。按照交通等基础设施先行的原则，实施“1123456”工程，重点推进路网、桥梁、港口、码头、水电等基础设施项目，全面整合沿江工业园区、港口码头、物流园区，着力构建以新区为核心的多式联运交通体系。力争今年一批工业项目和道路基础设施项目开工建设。积极推进港产城融合发展，加快文化教育、医疗卫生、餐饮商贸、住房保障、娱乐休闲等配套设施建设，完善综合服务功能。支持工业园区和城镇公共设施一体化建设，同步设计、共同推进，实现城园共建共享。重点推进来华管理区（来宾高新区）、河南工业园区、象州工业集中区、武宣工业园区产城互动试点建设。

注重成果转化做活科技创新。制定完善各项专业科技创新奖励扶持政策，推动“大众创业、万众创新”。组建市科技创新发展办公室和市新兴产业发展办公室，统筹推进科技创新和新兴产业发展。集中力量攻克一批制约产业发展的共性关键技术，加快推广应用一批新技术、新产品、新装备，组织企业实施重点领域工艺技术研究和新产品开发。实现科技成果转化10项以上，申报发明专利等知识产权力争突破100项，获得授权40项；新增高新技术企业3家；园区专业孵化器达5家，在孵企业达50家；建成2个以上综合创新平台。

三、着力推动提质增效，促进农业特色发展

以建设广西现代特色农业示范城市为目标，深入推进农业供给侧结构性改革，农林牧渔业增加值增长4.5%以上。

以优质供给为目标，全力优化产业产品结构。以实施现代特色农业“10+3”提升行动为载体，加快农业产业结构调整步伐。推进粮食生产功能区建设，良种覆盖率95%，粮食产量81万吨以上，抓好象州县作为自治区粮食主产区试点工作；新增糖料蔗10万亩、优质果园8万亩、桑园1万亩。着力发展秋冬农业综合开发和富硒农业。着重推广“微生物+”生态养殖技术，加快传统养殖业转型升级；大力发展名特优特色养殖；加强重大动物疫病防控工作，切实提高水产畜牧产品质量安全水平。加快调整林种结构，年内调减桉树1.5万亩。大力推行农业标准化生产和品牌建设，新增农产品“三品一标”认证10个以上；从源头减少农药、化肥使用，加强农产品质量安全体系建设，保障农产品安全有效供给。

以全产业链为导向，全力优化农业与二三产业深度融合。加快“两区三带”建设，推动产业融合发展。统筹布局优质稻、桑蚕、水果、生猪、肉牛等生产基地与农产品加工产业，推动糖料蔗综合利用、循环发展。鼓励企业发展农产品深加工，促进产业融合发展。打造20个农村一二三产融合发展示范村和“一村一品”示范村镇，新建成5个以上自治区级休闲观光农业与乡村旅游示范点。加快推进金凤凰农村产业融合发展先导区和“四季迁江•历史画廊”融农业、文化、旅游为一体的沿桥巩至迁江二级公路、红水河沿岸的现代休闲农业和文化旅游示范带建设。

以“四大工程”为重点，全力优化农业基础设施。加快推进桂中治旱、大藤峡水库移民安置、“双高”糖料蔗基地、现代特色农业（核心）示范区等重点工程，桂中治旱一期工程完成年度投资2亿元、二期工程完成5.5亿元；大藤峡水库移民安置工程完成投资10亿元；新增“双高”糖料蔗基地24万亩，总规模达64万亩；创建国家级示范区（产业园）1个以上，新建成自治区级现代特色农业（核心）示范区2个以上、市级4个以上、县级10个以上、乡级10个以上。统筹推进病险水库除险加固、中小河流治理等水利工程建设。加大土地开垦力度，加快高标准农田建设，坚守全市40.52万公顷耕地保有量和32.15万公顷基本农田保护红线。

以工业理念为主导，全力优化农业生产经营效益。实施新型农业经营主体“增量、扩容、提质”三大工程，积极发展农民专业合作社、家庭农场，创建一批示范农民合作社、示范家庭农场，指导培育新增农业企业10家以上，新增注册农民专业合作社200家以上、家庭农场100家以上，培养新型职业农民1000人以上。促进农业生产要素优化配置，大力实施农业规模化经营，扎实推进农村土地确权和农村产权交易平台建设，颁证率达95%以上。加强农业科技支撑作用，发展“互联网+”农业，推广测土配方施肥435万亩、节水农业150万亩。组建广西农业科学院来宾分院。主要农作物耕种收综合机械化水平达58%。

四、着力推动供需提升，促进服务业繁荣发展

以扩大消费为目标，加快服务业供给侧结构性改革，服务业增加值增长9.5%。

实施交通提升工程。交通固定资产投资额计划增长10%以上。以三江口港产城新区为重点，打造来宾西江综合交通运输体系。加快柳南高速改扩建来宾段、梧柳高速来宾段、柳州经合山至南宁高速来宾段建设，推进贺巴高速来宾段前期工作，其中蒙山至象州段争取2017年开工建设，加快县县通高速公路步伐。加快柳来河一体化发展进程，推动柳来工业大道来宾段开工建设，宜州至忻城高速公路列入规划；启动来宾至柳州城际铁路前期工作，推动柳广铁路经象州、武宣，柳州至韶关铁路经金秀尽早开工建设。推进乡乡通二级公路、屯屯通水泥路工程，建设二级公路300公里、三级公路100公里。推动港口和物流园建设，加快水运口岸建设，争取促成1至2个地方成为临时开放水域，提升港口货物运输和集散能力。

实施消费提升工程。推动实体零售店转型升级，培育限上企业净增10家。培育打造一批特色商业街，加大专业市场招商力度，搭建拉动消费新平台。继续开展百店大促销等活动，扩大消费需求。发挥“土专家”作用，发扬来宾“工匠精神”，培育打造兴宾大湾米粉、象州大米、武宣红糟酸、忻城糯玉米、金秀野生茶甜茶、合山坚果、来华凤凰葡萄、河南园迁江白砂糖等来宾品牌。改善农村消费环境，开拓消费市场。适应住房刚性需求和改善性需求，促进房地产业健康发展。大力发展电子商务，抓好电子商务进农村工作，引进农村淘宝服务站、邮乐购等消费新业态，力争电子商务交易额达40亿元以上。以创建“中国长寿市”为契机，着力发展健康养老产业，引进一批健康养生、医疗服务、老年人用品制造等项目，构建大健康产业基地。同时，加快金融、保险、文化、体育、家政、教育培训等服务业繁荣发展。

实施旅游提升工程。努力推动全域旅游，力争接待游客人数突破2000万人次，旅游总消费170亿元以上。加快推进推出金秀民宿旅游、象州特色古镇游、合山“记忆之城”、忻城薰衣草庄园升级改造、武宣大藤峡库区风光游、兴宾区“红河红”“清水清”城郊观光休闲特色旅游、来华管理区和河南工业园区“农业+旅游+文化”示范区、先导区等特色旅游。挖掘特色旅游资源，大力发展乡村旅游，打造独具特色的旅游品牌。提升红水河民族团结旅游带、大瑶山民族风情画廊品质，加大宣传推介营销力度，努力打造桂中旅游圈精品线路，积极融入周边城市大旅游圈。

五、着力推动项目建设和招商引资，夯实发展后劲

下大力推进重大项目建设。按照有目标、有线路图、有责任人、有时间表、有考核标准，完善重大项目推进工作机制，充分发挥市重大办协调监督和牵头抓总作用，加快296个重大项目建设，年度完成投资151亿元以上。加强项目协调管理，实现柳来工业大道一期、皇氏乳业乳制品加工基地等58个项目顺利开工；加快煤炭交易中心二期、水环境综合治理等101个续建项目建设；百悦商业广场等33个项目实现竣工。加强项目策划和储备，争取更多项目列入国家、自治区层面统筹推进。积极筹措项目建设资金，争取获得中央预算内投资和中央专项建设基金10亿元以上。创新投融资机制，加强筹融资工作，守住金融底线，通过PPP模式、政府购买服务、工程总承包、政府投资基金等渠道，为产业发展和城镇建设提供资金保障。把各县（市、区）、各部门争取的项目数和资金数作为重要考核指标，实行量化考核，压实责任。

下大力开展招商引资和开放合作。坚持把招商引资特别是招大商、招强商作为我市走出困境、跨越发展的重头戏和压舱石。擦亮和叫响“天下来宾，来者上宾”的品牌，充分利用国内区内多个区域合作平台载体的机遇，扩大开放合作，发展外向型经济。加强与北京市海淀区等国内外友好城市（城区）的合作交流。进一步完善招商引资项目库，突出抓好产业链、新兴产业、工业园区招商工作，着力“招大引强”，加快推进糖循环综合利用、生态铝精深加工、热电联产循环经济产业、合金新材料等传统产业“二次创业”招商；围绕碳酸钙、现代农机装备、茧丝绸、新材料、先进制造等新兴产业开展招商；围绕三江口港产城新区开展基础设施和产业配套招商。进一步改进完善招商引资的机制、模式、激励办法，营造招商引资良好氛围。力争全年招商引资规模超435亿元，增长10%以上，其中全口径实际利用外资1.5亿美元，努力实现10个以上投资超亿元产业项目落地开工。

六、着力推动脱贫攻坚，促进贫困群众脱贫奔小康

以更实的作风、更准的措施、更严的要求持续发力脱贫攻坚战，年内完成65个贫困村脱贫摘帽、4.1万以上贫困人口实现脱贫的目标任务。

扩大产业扶贫规模。结合农业产业结构调整，重点发展特色扶贫产业，支撑贫困户稳定增收。大力培育和引进龙头企业，充分发挥农民专业合作社、种植大户、电商网店等新型经营主体作用，辐射带动贫困群众增加收入。大力支持贫困村发展集体经济。设立扶贫信贷风险补偿资金，加快完善贫困地区信用和支付体系，撬动金融资本参与扶贫开发。加大政策扶贫力度，对能带动脱贫的产业、企业给予相应扶持。

推进易地扶贫搬迁。对建档立卡的贫困户和地质灾害区、生态保护区等需要整村整屯搬迁的农村群众，有计划的组织搬迁，力争年内搬迁9324户34110人，确保贫困群众搬得出、稳得住、能脱贫、可致富。

确保脱贫取得实效。切实抓好责任落实、政策落地和群众动员工作。以更实的作风开展扶贫，进一步细化、实化、具体化脱贫攻坚政策体系，扎实做好“一户一册一卡”和“双认定”工作。以更准的措施推动脱贫，按照“一户一策”要求，精准帮扶，精准施策，发挥群众主体作用，对贫困户做到底数清、情况明、数字准。以更严的要求强化扶贫资金管理，坚决防止弄虚作假、损害群众利益的“被脱贫”“假脱贫”“数字脱贫”现象发生。

七、着力优化城乡格局，促进区域协调发展

按照“大兴产业、完善提升、聚集人气、控制规模”的发展思路，推进城乡一体化，城镇化率达44.5%。

实施规划水平提升行动。完成城市总体规划修编工作，抓好社区服务设施等6项专项规划编制。加快完善控制性详细规划，开展城市风貌特色研究，做好传统镇、村落保护发展规划，不断提高规划科学水平。

实施城市承载力提升行动。主城区建成区面积达到46平方公里，城区人口达到35万人。继续开展城北新区配套服务功能完善提升工作，进一步完善基础设施建设，开工建设旧城区小街小巷道路提升改造、滨江北路西段等10个项目，完成8个项目建设，推进快速公交、智能公交和公共自行车交通服务项目建设。加快地下综合管廊、海绵城市等市政基础建设。抓好社区配套设施建设，提高社区服务能力。力争开工建设和改造提升市区（来宾高新区）至凤凰片区、市区（河南园东区）至迁江片区、市区至正龙片区三条城市大道，充分发挥凤凰片区、迁江片区、正龙片区作为城市副中心的功能作用，加快推进产城融合建设发展步伐。加快驻柳原地区三个大院的改造建设步伐。

实施城市管理提升行动。以创建全国文明城市为契机，加强市容市貌整治，提高市民文明意识。进一步理顺城市管理执法体制，提高执法水平。强化城市经营，整合盘活城市建设用地，创新城市建设投融资体制。推进智慧城市建设，促进城市管理智能化。

实施城镇化建设提升行动。加快推进新型城镇化综合试点建设，完善农业转移人口市民化配套政策，开展迁江华侨农场棚户区改造二期等7个棚户区改造工作。推进象州县、武宣县两个国家级改革试点工作，加强特色名镇名村建设。

实施生态宜居环境提升行动。严格执行“水十条”“大气十条”“土十条”规定，落实“河长制”，守住“天上、山上、水里、地里”四条生态底线，开展“美丽来宾•宜居城市”创建活动，构建山清水秀的自然生态。加强城市绿化美化建设，在红水河沿岸市区段、柳南高速市区段、高铁市区段以及“桂中水城”堤岸、城市主干道两旁集中种植木棉树，大力营造来宾特色木棉绿化景观带，今年种植木棉树2万株以上；继续完善提升“桂中水城”建设，打造具有来宾地方特色的“红河红—木棉花城”和“清水清—桂中水城”。改造完善提升凤凰山森林公园、老梅沟森林公园、蓬莱洲森林公园、北合盘古文化森林公园、金龟岛生态休闲公园等城郊生态休闲景区，打造城市生态宜居的“绿肺”和休闲旅游的新名片。完成造林面积22万亩，打造“国家园林城市”，巩固“国家森林城市”成果。支持金秀县创建国家重点生态功能区。大力推广清洁能源，加强工业废气、建筑粉尘、道路扬尘等综合整治，空气质量优良天数超过90%。加快推进污水垃圾处理设施建设。创建国家新能源示范城市，完成自治区下达的节能减排目标任务。完善环境监管机制，落实“党政同责，一岗双责”。以实施“产业富民、服务惠民、基础便民”三个专项活动为抓手，集中开展“美丽来宾•宜居乡村”活动，全力打造“美丽来宾”乡村建设升级版。

实施县域经济实力提升行动。各县（市、区）、来华管理区（来宾高新区）、河南工业园区要高度重视县域产业的发展壮大，在抓好一产、三产加快发展的同时，要充分发挥自身优势，更加聚焦发展工业，做大做强工业，全力提升经济实力。兴宾区要加快三江口新区高安片区、正龙片区、小平阳片区的工业园区开发建设，加快糖业、碳酸钙产业的发展，实现生产总值增长7.5%以上。象州县要以三江口新区石龙片区、马坪片区建设为契机，打造特色产业，大力引进和发展新能源、汽配加工等新兴产业，推进染整业项目尽快落地，实现生产总值增长7.5%以上。武宣县要着力打造碳酸钙产业升级园，尽快启动三江口新区金鸡片区、黄茆片区建设，大力发展碳酸钙等新兴产业、特色经济、低碳经济，实现生产总值增长7.5%以上。忻城县要继续大力发展石材、茧丝绸产业，实现生产总值增长8%以上。金秀县要大力发展“山下”生物医药、农产品深加工等特色产业，加快推进三江口新区桐木、头排拓展区的开发建设，实现生产总值增长7.5%以上。合山市要巩固提升生态火电、热电一体和加快碳酸钙等产业的发展，实现生产总值增长8%以上。来华管理区（来宾高新区）和河南工业园区以高新技术产业、先进制造业、冶炼业、热电联产、铝精深加工为重点，聚焦工业、高新技术产业，挺起我市工业发展的脊梁，规模以上工业增加值分别增长13%、8%以上。

“天上不会掉馅饼，努力奋斗才能梦想成真。”发展犹如逆水行舟，不进则退，慢进也是退。六个县（市、区）、来华管理区（来宾高新区）、河南工业园区以及各产业管理职能部门都要高度重视各项主要经济指标任务的落实完成，不得以各种理由、各种形式怠慢、推脱和不作为、慢作为。作为后发展落后地区的县市区、园区，更不能使指标下降或完不成指标任务成为“习惯”。我们要的是通过主动作为、克难攻坚、担当尽责，通过实干苦干、拼命干、撸起袖子加油干，实实在在干出来、做出来的更快更好的指标，使我市的经济指标在全区排位从末位尽快提升靠前，为我市经济尽快走出困境多作担当、多作贡献。年终对县（市、区）、来华管理区（来宾高新区）、河南工业园区和各相关主管责任部门的绩效考核，不仅要全面考核各项主要经济指标的完成情况，还要进一步加大考核的权重，把绩效考核与个人绩效、评先评优相挂钩，要通过全市上下齐心努力，共同营造一个“做大产业、做强工业、做精招商、做实项目”的浓厚氛围。

八、着力保障民生底线，营造团结和谐的社会生态

保障民生是政府工作的硬底线，维护稳定是硬任务，必须统筹推进，筑牢基础。

加快教育改革发展。加强师资队伍建设，多措并举提升教育教学质量。扩大城乡学前教育资源，加快普及学前三年教育，学前三年毛入园率达85%。统筹推进县域义务教育城乡一体化改革发展，确保金秀县、兴宾区义务教育均衡发展通过国家评估认定，义务教育巩固率达94%。加快推进城区普通高中规划建设，来宾实验高中迁建项目投入使用，启动柳州地区高中迁建来宾项目建设；继续推进中等职业教育综合改革，高中阶段毛入学率达89%。继续支持广西科技师范学院加快发展，全面完成来宾职业技术学院的组建工作，加快推进高等教育转型发展。

提升文化服务水平。完善国家基层综合性文化服务中心建设，不断提高广播电视有效覆盖，扎实推进城北新区公共文化设施建设，市图书馆、档案馆、群众艺术馆建成并投入使用。鼓励文艺精品创作，大力发展文化产业，文化产业增加值占生产总值1.5%以上。加强文化遗产保护利用，继续做好“麒麟山”“世界瑶都”等壮瑶文化的挖掘和保护开发。加大“扫黄打非”力度。大力开展各类群众文体活动，举办庆祝来宾市成立15周年系列体育赛事和群众文娱活动，巩固提升全国“全民健身示范城市”工作成果。

完善社会保障体系。稳步推进机关事业单位养老保险制度改革及异地就医、大病保险等改革工作，统筹推进城乡居民社会养老保险制度；新增城镇就业8250人以上，农村劳动力转移就业新增2.7万人次以上。探索农村五保供养对象分类分级管理，进一步提高城镇、农村居民的最低生活保障标准。加快住房保障体系建设，建设保障性住房1.3万套，完成农村危旧房改造6000户。采取有效措施破解拖欠农民工工资问题。

提升健康保障服务水平。加快市级、县级公立医院改革，构建高质量的医疗服务体系和分级诊疗格局；全力推进基层医疗卫生机构能力建设，加大基层医疗人才培养力度，增加基层医疗资源供给，不断提高基层医疗卫生体系服务能力；扎实开展中医药、壮瑶医药健康服务和幸福家庭创建工作。提升计划生育服务水平。

加强食品药品监管和安全生产。强化食品药品基层基础，增强监管力度，支持武宣县争创国家农产品质量安全县，扎实推进食品安全城市创建。固守安全生产底线，健全安全生产责任体系、隐患排查治理体系、事故防控体系和应急救援体系，实现全市安全生产事故总量、死亡人数持续下降，杜绝重大事故发生，为全市经济发展提供安全保障。

坚守社会和谐稳定底线。加强法治宣传和法律援助工作。完善社会风险防控机制建设。扎实做好信访维稳和矛盾纠纷排查化解工作。夯实社区基础建设和保障机制，全面推进社区网格化服务管理。打好禁毒人民战争。创新社会治安综合治理，开展“综治中心建设年”活动，不断完善立体化防控体系，为党的十九大胜利召开营造和谐稳定的社会环境。进一步加强军民融合发展，继续创建全国双拥模范城。

切实抓好为民办实事工作。在完成自治区为民办实事工作基础上，实施我市十件为民办实事工程。

九、着力提升服务环境，形成融合发展合力

深化重点领域改革。全面落实中央和自治区的各项改革任务，做到上级有要求，来宾有行动，行动有成效。完成好中央和自治区赋予来宾的试点改革任务，发挥好先行先试的作用。加大来宾的“自选改革”力度，以解决核心问题为导向，聚焦突破瓶颈，继续完善“三区”管理体制改革、三江口新区开发建设体制、重大项目建设机制、农村综合改革、绩效考核奖励机制等改革。适应我市“做大产业、做强工业、做精招商、做实项目”的新形势新要求，进一步深化市管企业、平台公司改革改制，健全完善国有资产监管体系。以提高效率为目标，深化行政审批制度改革，进一步抓好简政放权、放管结合、优化服务，推进行政审批服务事项目录和标准化建设，建成一体化网上政务服务平台；加强信用体系建设；提高公共资源交易电子化服务水平。通过改革，有效激发市场活力和社会创造力。

营造风清气正的政治生态。积极推进法治政府建设，坚决维护市委的领导核心，自觉接受市人大及其常委会的法律监督、市政协的民主监督和社会的舆论监督；坚持依法行政，严格公正文明执法，切实维护群众合法权益；提高行政决策科学化、民主化、法治化水平。要坚持“守底线，用干部，善作为”，坚持务实担当，敢于直面问题，敢于触及矛盾，善于破解难题，把责任扛在肩上，要勇于挑最重的担子，啃最硬的骨头，做到不回避、不推诿、不退缩；突出执行力、贯彻力、落实力，做到接地气、聚人心，把政府工作的成效、实效真正体现在执行落实的能力和水平上。坚持开拓创新，勇于突破传统、大胆尝试、创先争优，不断激发干部的工作热情和积极性，杜绝“为官不为”现象，进一步提升政府的创造力、凝聚力和战斗力。坚持廉洁从政，严守纪律底线，认真落实全面从严治党主体责任，按照法定权限和程序履职尽责，切实把党风廉政建设各项要求落到实处；严格落实中央八项规定精神，持之以恒改进作风，实现干部清正、政府清廉、政治清明。

各位代表！历经风雨才能见到彩虹，唯有奋斗才能赢取未来。让我们更加紧密地团结在以习近平同志为核心的党中央周围，在自治区党委、政府和市委的坚强领导下，着力营造“三大生态”，发扬“心容天下，敢为人先”的来宾精神，咬定目标，坚韧不拔，脚踏实地，埋头苦干，努力完成今年经济社会发展目标任务，为建市15周年献计出力，以优异的成绩迎接党的十九大胜利召开！
